# Household physical activity is positively associated with gray matter volume in older adults

**DOI:** 10.1186/s12877-021-02054-8

**Published:** 2021-02-05

**Authors:** Noah D. Koblinsky, Liesel-Ann C. Meusel, Carol E. Greenwood, Nicole D. Anderson

**Affiliations:** 1 Rotman Research Institute, Baycrest Hospital, Toronto, ON Canada; 2 Toronto Rehabilitation Institute, University Health Network, Toronto, ON Canada; 3 Department of Nutritional Sciences, Faculty of Medicine, University of Toronto, Toronto, ON Canada; 4 Departments of Psychology and Psychiatry, University of Toronto, Toronto, ON Canada

**Keywords:** Household physical activity, Brain volume, Cognition, Older adults

## Abstract

**Background:**

Total physical activity is positively associated with brain volume and cognition in older adults. While we have ample evidence that recreational physical activity influences brain health, the contributions of other daily activities are less understood. In particular, the associations between household physical activity and brain health in older adults is underexplored. The purpose of this study was to identify associations between household physical activity, brain volume, and cognition in a sample of cognitively unimpaired older adults.

**Methods:**

We report data from 66 cognitively unimpaired older adults (71 ± 4 years) who participated in a health evaluation, cognitive assessment, and structural brain imaging. Physical activity was assessed using the Phone-FITT questionnaire and separated into household and recreational physical activity. We quantified whole brain volume, gray matter volume, and white matter volume, and assessed cognitive performance in four domains: memory, working memory/attention, processing speed, and executive function. Associations between physical activity, brain volume, and cognition were investigated in an omnibus approach using two multivariate analysis of variance (MANOVA) models. The first model assessed the associations between physical activity and brain volume adjusting for age, sex, Framingham Risk score (FRS) and intracranial volume. The second model assessed the associations between physical activity and overall cognitive performance adjusting for age, sex, FRS and education. Post hoc regression analyses were conducted to investigate significant MANOVA results. We also conducted further regression analyses to investigate associations with hippocampal and frontal lobe volume.

**Results:**

Household, but not recreational, physical activity was positively associated with brain volume measurements (*F* = 3.07, *p* = .035), specifically gray matter volume (t = 2.51, *p* = .015). Further exploratory analyses identified that household physical activity was associated with hippocampal (*p* = .015) and frontal lobe (*p* = .010) volume. No significant relationships were observed between household or recreational physical activity and cognition.

**Conclusion:**

Time spent engaging in household physical activity was positively associated with brain volume, specifically gray matter volume, in older adults. Highlighting the benefits associated with household chores may motivate older adults to be more active by providing a more attainable, low risk form of physical activity.

## Background

Alzheimer’s disease and related dementias top the world’s most prevalent and costly medical conditions [1] and prevention and treatment of these disorders has been deemed a public health priority by the World Health Organization [2]. It has become widely accepted that unhealthy lifestyle behaviours contribute to an increased prevalence of cognitive impairment and dementia worldwide [3] and that engaging in physical activity is an effective strategy for preserving brain health in older adults [4–8]. Broadly defined, physical activity is any activity that involves bodily movements and the use of skeletal muscles. This includes everyday activity that is not structured, such as occupational or household activity, as well as recreational physical activity, such as exercise, which is structured and intended to improve fitness [9]. The actions by which physical activity affect the brain are suspected to be both indirect, such as improving health conditions, as well as more direct, including increasing brain neurotrophins [6, 10], improving cerebrovascular function [11, 12], and enhancing brain plasticity [13, 14]. Understanding how different forms of physical activity (i.e., recreational activity vs. household activity) contribute to brain health is crucial for developing strategies to reduce the risk of cognitive decline and dementia in older adults.

Much of the research in this regard has focused on recreational physical activities, in particular the effects of higher intensity exercise. Moderate to vigorous intensity exercise and higher aerobic fitness have been positively associated with whole brain volume, gray matter volume, and improved white matter integrity [5, 6, 15–19]. Moreover, exercise interventions have demonstrated effectiveness at increasing whole brain volume and gray matter volume in older adults [20–22]. Similar findings are also evident with low intensity forms of exercise independent of moderate to vigorous intensity activity [23–26]. A comprehensive review of the literature by Erickson et al. (2014) concluded that physical activity and fitness levels were routinely associated with larger hippocampal and prefrontal cortex volumes, and that exercise interventions were able to evoke positive changes in these regions specifically [6]. Cognitive function in older adults is also positively associated with cardiovascular fitness and in some studies has been shown to improve following exercise interventions [4, 27] with greatest benefits being apparent in executive function and attentional control. Not all studies show this effect, however. For example, a recent review of physical activity interventions concluded that evidence for cognitive improvement following interventions was insufficient, largely due to the heterogeneous cognitive measures used across studies, and underpowered sample sizes [28].

We know that not all brain benefits are derived from recreational physical activity exclusively. Total physical activity is associated with reduced cognitive decline and dementia risk [29–31], decreased brain atrophy [32], as well as increases in brain volume [33–36]. While ample evidence exists that recreational physical activity contributes to these associations, less is known about how other everyday physical activities such as household activity contribute to brain health. Household activities may be particularly interesting to study, because these types of activities (e.g., cleaning chores, meal preparation) are inherent aspects of many people’s daily life, providing some degree of physical activity and decreasing sedentary behaviour naturally. Studies examining the role of household physical activity on brain health are limited, however. Engagement in household physical activity has been shown to be negatively associated with frailty [37] and leisure time activity incorporating household chores has been found to be associated with decreased dementia risk [38, 39]. The purpose of this study was to assess how household physical activity specifically correlates with brain volume and cognition among a sample of cognitively unimpaired older adults. We hypothesized that household physical activity would be positively associated with both brain volume and cognition.

## Methods

### Study design and overview

Participants attended three assessment visits at Baycrest Hospital in Toronto involving a health evaluation, cognitive assessment, and structural brain imaging. Additional demographic, medical, and cognitive screening questions were administered by telephone before individuals were invited to participate. A physical activity questionnaire was administered to assess participants’ levels of household and recreational physical activity. The work described was carried out in accordance with The Code of Ethics of the World Medical Association (Declaration of Helsinki) for experiments involving humans and was approved by the Research Ethics Board at Baycrest. All participants provided written informed consent.

### Participants

We recruited 70 cognitively unimpaired older adults through advertisements and the Baycrest research participant pool. Participants were required to be 65–85 years of age, cognitively unimpaired and free from significant medical, neurological, or psychiatric conditions. Participants were excluded from entering the study if they met any of the following criteria: a score < 30 on the Telephone Interview for Cognitive Status– modified version (TICS-m) [40] to exclude individuals with possible dementia; use of insulin to treat type 2 diabetes; self-report of major diabetic complications, such as gastroparesis, retinopathy, nephropathy, or neuropathy; other significant medical or mental health disorders affecting cognition, such as previous myocardial infarction, stroke (self-report or evident from structural scans), or major depressive disorder; current or recent use of central nervous system-active medications, including those for the treatment of depression, sleep disorders, and migraine headaches; major inflammatory disorders, heart failure, and chronic lung disease; hormone replacement therapy in female participants; or contraindications to magnetic resonance imaging (e.g. claustrophobia, obesity, implanted metal from a surgery or injury). Two participants were removed from the analysis as outliers, one for presenting with a low intracranial volume (2.7 standard deviations below the mean) and the other for having a high FRS value (4.3 standard deviations above the mean). An additional two participants did not complete the cognitive test battery. Our final analysis includes 66 participants.

### Health evaluation

Resting blood pressure was assessed (BPTru Medical Devices) and the average of four seated measurements was recorded. A fasting blood sample was collected for measurement of triglycerides, cholesterol (total, low density lipoprotein [LDL], and high density lipoprotein [HDL]), glucose, insulin, and HbA1c. Cardiovascular burden was assigned using the Framingham Risk Score (FRS). FRS was calculated using the Cox model formula [41] which includes age (years), sex (male/female), treatment of systolic blood pressure (yes/no), systolic blood pressure (mmHg), total cholesterol (mg/dL), HDL (mg/dL), smoking (yes/no), and diabetes mellitus (yes/no). FRS estimates each participant’s probability of developing cardiovascular disease (including cerebrovascular events) over a 10-year period [42]. Participants were asked to continue their usual diet, medications, and physical activity for the remainder of their involvement in the study.

### Cognitive assessment

All participants were administered a battery of neuropsychological tests comprising four domains; memory, attention/working memory, executive function, and processing speed. Memory tests included the California Verbal Learning Test II immediate and delayed recall [43], Wechsler Memory Scale-Revised (WMS-R) Visual and Verbal Paired Associates learning and delayed recall [44], and the Wechsler Memory Scale-III (WMS-III) Faces immediate recall [45]. Attention/working memory tests included the WMS-III Mental Control [45], WMS-III Letter-Number Sequencing [45], Wechsler Adult Intelligence Scale-III (WAIS-III) backwards Digit Span [46], WAIS-III forward Digit Span [46], and Wechsler Adult Intelligence Scale – Revised (WAIS-R) Arithmetic [47]. Executive functioning tests included the Wisconsin Card Sorting test – Modified (number of errors) [48], Controlled Oral Word Association (COWA) test - Phonemic fluency (FAS) [49], and the Halstead-Reitan Trail Making Test version B [50]**.** Processing speed tests included the Halstead-Reitan Trail Making Test version A [50] and WAIS-III Digit Symbol Coding [46]. Neuropsychological test scores are reported as raw scores. Composite scores for each domain were created by calculating z scores for each test relative to the current sample (*n* = 66) and averaging them.

### Structural imaging

Images were acquired on a 3 Tesla Magnetom Trio Siemens scanner with a 12-channel head coil. Each participant’s head was restrained using a vacuum pillow that fit inside the head coil. High-resolution structural images (T1-weighted three-dimensional magnetization-prepared rapid gradient-echo sequence; 3D-MPRAGE) were acquired with the following parameters: TR/TE = 2000/2.63, FOV = 256 mm, slice thickness = 1 mm, number of slices = 160. Cortical reconstruction and volumetric segmentation on the native-space anatomical images were performed with the Freesurfer image analysis suite (version 5.3.0; http://surfer.nmr.mgh.harvard.edu/). Before data extraction, an in-house quality assurance protocol was implemented based on recommended procedures from the Freesurfer website. Briefly, each brain was assigned a rating of 1, 2, or 3 based on the overall quality of the output. Brains with few, minor issues (e.g., tiny inclusions of skull/meninges in the pial surface, minute errors in labeling the white/gray matter interface) were assigned a rating of 3 (*n* = 53). Brains with more frequent or slightly larger errors were assigned a rating of 2 (*n* = 13), and brains with the most issues were assigned a rating of 1 (*n* = 0). Within the present sample there were no brains with errors considered substantial enough to require manual correction or exclusion. For analyses, we then extracted estimates of intracranial volume, whole brain volume, gray matter volume, and white matter volume. Hippocampal and frontal lobe volumes were also extracted, using the Desikan-Killiany atlas [51]. To account for variability in head size in our analyses, all of the brain volumetric measures were adjusted for intracranial volume (ICV) [52].

### Physical activity

Physical activity was assessed using the Phone-FITT, a valid and reliable telephone questionnaire for community dwelling older adults which gathers information on frequency, duration, and intensity of all physical activities performed over the past month [53]. The Phone-FITT questionnaire can be found in Gill et al. [53]. The questionnaire accounts for seasonal variance, allows individuals to report on activities not specifically listed, and separates activities into household and recreational physical activity. Household physical activity includes: light housework (tidying, dusting), meal preparation/clean up, shopping, heavy housework, home maintenance (e.g., yard work and home repairs), and care giving. Recreational physical activity includes: arm strengthening, leg strengthening, stretching and balance exercises, walking for exercise, dancing, swimming, bicycling, golf, gardening, and any other activities reported by the individual. Frequency is measured as number of times the activity was performed per week and duration for each activity is scored categorically as 1 (1 to 15 min), 2 (16–30 min), 3 (31–60 min), or 4 (60+ minutes). Intensity is measured by asking respondents whether they were breathing normally, slightly out of breath, or breathing too hard to carry on a conversation (scored as 1, 2, and 3, respectively) when performing the activity. The authors suggested that frequency and duration scores be used exclusively [53] as intensity measures tend to decrease response reliability. Within our sample, we found that household activities done multiple times per day (e.g., preparing meals, tidying) overinflated total scores so we chose to analyze the duration scores only. Therefore, the scores used are the sum of duration scores from each activity regardless of frequency or intensity, and hereafter are referred to as household and recreational physical activity.

### Analyses

We performed statistical analyses using R (v.3.3.1) [54]. Significance was set at *p* < .05. First, we examined the effect of potential confounding variables of age, education, and FRS on brain volume and cognition using Pearson correlation. FRS was included as a covariate in our models due to the abundance of research indicating that FRS is negatively associated with brain structure and function in cognitively unimpaired older adults [55]. Relationships with brain volume measurements were assessed by partial correlation adjusting for ICV. T-tests were used to assess sex differences in household and recreational physical activity scores. Sex differences between brain volumes and cognition were assessed using analysis of covariance (ANCOVA) and adjusted for age, FRS, and ICV in brain volume analysis, and age, FRS, and education in cognition analysis.

We tested the relationships between physical activity, brain volume, and cognition in an omnibus approach using two multivariate analysis of variance (MANOVA) models [56]. Household and recreational physical activity scores were entered together as independent variables in both models. The dependent variable in the first model was brain volume, which was comprised of 3 measurements: whole brain, gray matter, and white matter volume. The dependent variable in the second model was cognition, which was comprised of composite z scores for four cognitive domains: memory, attention/working memory, executive function, and processing speed. Age, sex, FRS, and intracranial volume were used as covariates in the model examining brain volume. Age, sex, FRS, and education were used as covariates in the model examining cognition. Post-hoc multiple regression models were used to further explore dependent variables from significant MANOVA models. The Bonferroni method was used to adjust for multiple comparisons. Statistical threshold for significance was set at <.017 for individual brain volumes (.05/3) and < .0125 for individual cognitive domains (.05/4). Due to the well-established literature associating hippocampal and frontal lobe volume with physical activity [6], we also conducted exploratory regression analyses to investigate these relationships in our sample.

## Results

Table 1 describes the demographic, neuroimaging, and cognitive characteristics of the study sample.

**Table 1:** Participant characteristics

**Demographics**	
Age (years)	71 ± 4 (65–83)
Sex (count)	41 Females (62%)
Education (years)	16 ± 3 (10–23)
Framingham Risk Score	.17 ± .10 (.03–.37)
Household Physical Activity - Phone-FITT Score	10.67 ± 4.28 (1–20)
Recreational Physical Activity - Phone-FITT Score	9.39 ± 5.40 (0–21)
**Neuroimaging**	
Intracranial Volume (cm^3^)	1487 ± 171 (1090 – 1862)
Whole Brain Volume (cm^3^)	1066 ± 111 (799–1311)
Gray Matter Volume (cm^3^)	569 ± 53 (429–672)
White Matter Volume (cm^3^)	434 ± 58 (323–584)
**Cognition**	
Memory Domain	
CVLT - Learning	49.76 ± 9.58 (29–69)
CVLT - Delayed Free Recall	10.98 ± 3.16 (0–16)
Visual Paired Associates - Learning	12.58 ± 3.59 (5–18)
Visual Paired Associates – Delayed Free Recall	5.53 ± 0.93 (2–6)
Verbal Paired Associates - Learning	17.92 ± 3.14 (10–24)
Verbal Paired Associates – Delayed Free Recall	7.09 ± 1.06 (5–8)
Faces – Immediate Recall	35.53 ± 3.90 (28–45)
Attention / Working Memory	
Digit Span Forwards	10.97 ± 2.47 (5–16)
Digit Span Backwards	7.68 ± 2.52 (3–14)
Letter – Number Sequencing	10.62 ± 2.68 (4–17)
Arithmetic	12.67 ± 3.23 (6–19)
Mental Control	25.11 ± 4.95 (15–38)
Executive Function	
WCST errors	22.12 ± 12.75 (7–52)
Trail Making Test – Version B	82.82 ± 29.35 (36–173)
Phonemic Fluency (FAS)	43.55 ± 12.40 (23–74)
Processing Speed	
Digit Symbol Coding	61.11 ± 12.95 (28–91)
Trail Making test – Version A	36.48 ± 11.71 (19–87)

Continuous data are presented as mean ± SD (range) and categorical data are presented as count (percentage). Phone-FITT scores are the sum of duration scores for household and recreational categories. Cognitive data are unadjusted mean ± SD (range) raw test scores; CVLT = California Verbal Learning Test; WCST = Wisconsin Card Sorting Test. *N* = 66

Bivariate correlations between variables are displayed in Table 2. Age was negatively associated with gray matter volume (*r* = −.40, *p* < .001). Years of education was positively associated with attention/working memory (*r* = .33, *p* = .006) and executive function (*r* = .39, *p* = .001). FRS was negatively associated with whole brain (*r* = −.28, *p* = .026), white matter volume (*r* = −.28, *p* = .024), memory (*r* = −.39, *p* = .001), and executive function (*r* = −.30, *p* = .015). There were no significant sex differences in physical activity or brain volume measurements; however, memory performance was better in women (*p* < .001).

**Table 2:** Bivariate correlations

	Age	Education	FRS	Household Physical Activity	Recreational Physical Activity	Whole Brain Volume	Gray Matter Volume	White Matter Volume	Memory	Working Memory	Executive Function	Processing Speed
Age	1											
Education	0.01	1										
FRS	0.18	−0.11	1									
Household Physical Activity	−0.18	− 0.23	−0.11	1								
Recreational Physical Activity	0.04	0.01	−0.15	0.19	1							
Whole Brain Volume	−0.24	−0.15	−0.28^a^	0.36^b^	0.19	1						
Gray Matter Volume	−0.40^c^	−0.07	− 0.22	0.36^b^	0.16	0.71^c^	1					
White Matter Volume	−0.07	−0.17	−0.28^a^	0.27^a^	0.15	0.80^c^	0.23	1				
Memory	−0.06	0.12	−0.39^b^	0.10	0.13	0.36^b^	0.19	0.26^a^	1			
Working Memory	−0.04	0.33^b^	−0.03	0.00	0.18	0.17	0.15	0.08	0.21	1		
Executive Function	−0.20	0.39^b^	−0.30^a^	− 0.14	0.19	0.35^b^	0.35^b^	0.23	0.35^b^	0.57^c^	1	
Processing Speed	−0.13	0.24	−0.05	0.08	0.14	0.31^a^	0.25^a^	0.17	0.35^b^	0.44^c^	0.61^c^	1

FRS = Framingham Risk Score; Partial correlations adjusting for intracranial volume were used to assess brain volume, ^a^ < .05, ^b^ < .01, ^c^ < .001. *N* = 66

The MANOVA revealed that household physical activity (*F* (3, 57) = 3.07, *p* = .035), but not recreational physical activity (*F* (3, 57) = 0.32, *p* = .812) was significantly associated with brain volume measurements. Age (*p* = .043), sex (*p* = .003), and FRS (*p* = .025) were significant covariates in this analysis. Neither household (*F* (4, 56) = .1.52, *p* = .210) nor recreational physical activity (*F* (4, 56) = .74, *p* = .571) were associated with overall cognitive performance, but women had better cognitive performance (*p* < .001). Table 3 displays the results from the post hoc multiple regression analysis. Household physical activity was positively associated with gray matter volume (*p* = .015) and age was the only significant covariate (*p* = .008).

**Table 3:** Associations between physical activity and individual brain volume measurements

	Whole Brain Volume			Gray Matter Volume			White Matter Volume		
	b (SE)	t	***p***	b (SE)	t	***p***	b (SE)	t	***p***
Household Physical Activity	2.82 (1.17)	2.41	.019	2.17 (0.86)	2.51	.015	1.82 (0.99)	1.85	.070
Recreational Physical Activity	0.82 (0.92)	0.90	.371	0.46 (0.66)	0.69	.495	0.39 (0.77)	0.50	.616

Models adjusted for age, sex, FRS, and ICV; b = unstandardized parameter estimate; SE = standard error; t = t score; *p* = significance value. *N* = 66

Exploratory regression analyses revealed that household physical activity was significantly associated with hippocampal (*p* = .015) and frontal lobe (*p* = .010) volumes (Table 4). To investigate the lack of relationship between recreational physical activity and brain volume, an additional analysis was conducted excluding light intensity activities (e.g., light walking, stretching/balance exercises, gardening, golf) from our recreational measure. As with the previous analyses, recreational physical activity was not associated with gray (*p* = .598) or white (*p* = .149) matter volumes.

**Table 4:** Associations between physical activity, hippocampal volume, and frontal lobe volume

	Hippocampal Volume			Frontal Lobe Volume		
	b (SE)	t	p	b (SE)	t	p
Household Physical Activity	2.17 (0.86)	2.51	.015	7.15 (2.67)	2.68	.010
Recreational Physical Activity	0.46 (0.66)	0.69	.495	1.76 (2.09)	0.84	.403

Models adjusted for age, sex, FRS, and ICV; b = unstandardized parameter estimate; SE = standard error; t = t score; *p* = significance value. *N* = 66

## Discussion

This study investigated the associations between household physical activity, brain volume, and cognition in a sample of cognitively unimpaired older adults. These results supported our hypothesis that household physical activity is positively associated with brain volume, however no significant associations with cognition were observed. The overarching association with brain volume was driven by gray matter volume, but not white matter volume. We believe that this was reflected in the trend towards a significant association with total brain volume, however this association failed to reach statistical significance after Bonferroni correction. The hippocampus and frontal lobe have been identified as brain areas that are particularly sensitive to exercise [6] and our work supports this notion in the context of household physical activity. Surprisingly, no associations were observed between recreational physical activity and brain volume or cognition.

This is the first study to identify an association between household physical activity and gray matter volume, and contributes to the growing body of research helping to guide physical activity recommendations for older adults. Our work uniquely points to an association with previously unexplored components of total physical activity. While there is ample evidence for the benefits of recreational physical activity, we now show how a specific type of activity that is an inherent aspect of many people’s daily life relates to brain health. Highlighting the brain benefits associated with household chores (e.g., cooking, cleaning, home maintenance) may motivate older adults to be more active by providing a more sustainable, low risk form of physical activity.

It is commonly hypothesized that increases in brain volume result from improved fitness and enhanced blood flow following exercise [11]. Total physical activity is associated with enhanced cardiorespiratory fitness and decreased incidence of cardiovascular disease and mortality [57]. It has been suggested that the vascular effects from non-structured everyday activities may be similar to those resulting from low intensity aerobic exercise [58]. For example, Sanchez-Lopez et al. [59] observed positive associations between non-structured physical activity and brain activity, and posited that results may have been due to vascular mechanisms based on hematological differences between study subjects. Another potential mechanism linking household physical activity to brain volume is enhanced neuroplasticity resulting from the planning and organization required for completing household chores. Interventions focusing on goal management and multitask training have shown to be effective at improving brain function in older adults [60–63]. Housework includes a wide range of tasks and may share many of the same features as cognitive training interventions [64]. On the contrary, it may be that individual's brain volume influences their level of household physical activity engagement. For example, someone experiencing greater than normal atrophy may be less likely to engage in household tasks. Lastly, an intriguing consideration is that the increased household physical activity scores in our sample may reflect lower levels of sedentary behavior. Approximately 67% of older adults report sitting for more than 8.5 h per day [65] and sedentary time is associated with adverse health outcomes despite regular exercise engagement [66, 67]. Prolonged sitting increases venous pooling and coagulation factors which in turn disrupt blood flow [68] and can lead to spikes in blood glucose and plasma triglycerides that are detrimental to the vasculature [69]. Sedentary behavior is associated with brain atrophy [29, 70] and research suggests that replacing sedentary time with light activity may promote optimal brain health [71–73]. We did not collect measures of sedentary behavior in this study but is an important measure to consider in future trials exploring the link between household physical activity and brain volume.

We proposed that our main results may be explained by the cognitive involvement of household chores, however physical activity was not associated with cognition in our sample. It is possible that more sensitive measures of planning and multitasking are required to confirm this association. Not all research supports the link between physical activity and cognition. A recent systematic review by Brasure et al. [28] concluded that evidence of a link between physical activity and dementia prevention is insufficient. The authors acknowledged limitations of the existing studies, including heterogeneous protocols and cognitive measures, as well as underpowered sample sizes. We may not have had sufficient power to observe the relationship between physical activity and cognition that has been reported in the literature [27–31]. While associations between physical activity and cognition were not apparent in our sample, brain volumetric measurements are strong predictors of longitudinal cognitive change [74, 75].

The lack of association between recreational physical activity and indicators of brain health in our sample was unexpected, but is perhaps explained by the removal of intensity scores from the analysis. We chose not to analyze intensity scores as per the recommendations of the Phone-FITT authors over concerns about the reliability of self-reported exercise intensity. Furthermore, although Phone-FITT recreational physical activity scores incorporate moderate to vigorous intensity physical activities, several low intensity activities such as gardening, golf, tai chi, stretching, and balance exercises are included in the questionnaire. The inclusion of these activities may have attenuated associations between recreational physical activity, brain volume, and cognition. Exploratory analyses were conducted on our dataset by removing light intensity activities (light walking, stretching, golf, and gardening) however these analyses did not lead to significant changes to our results. The removal of intensity scores may also help to explain the lack of associations with white matter volume. White matter abnormalities are a hallmark sign of cerebral small vessel disease, a condition that is attributable to poor cardiovascular health [3]. It is likely that white matter volume would show greater associations with participation in higher intensity exercise aimed at improving cardiovascular health and fitness. When light intensity activities were excluded form recreational physical activity scores, a closer association was observed between recreational physical activity and white matter volume, however, it still failed to reach significance.

As the first study to explore the relationships between household physical activity and brain volume, we first investigated more general areas of the brain, but conducted exploratory analysis to reveal regional associations. Consistent with the literature, associations were observed between hippocampal and frontal lobe volume. Further work with larger samples would allow for more precise characterizations of areas of the brain associated with household physical activity. Furthermore, while we proposed several mechanisms that may explain our results, we were unable to test these notions statistically. We did not collect measures of sedentary behaviour or quantify the cognitive involvement relating to household physical activity. Although we did calculate cardiovascular burden (FRS), larger studies with higher power are needed to test how cardiovascular health mediates the relationship between household physical activity and brain volume. It would be a worthwhile objective for future studies to investigate these potential mechanisms as mediators.

Like all self-report measures, the Phone-FITT is prone to social desirability and recall bias. Using the previous month as the recall period may avoid some of the difficulty with recalling behaviour over longer time periods but it is still likely that individuals feel the need to overestimate healthy behaviors. The Phone-FITT also provides an arbitrary score that cannot be translated to common physical activity parameters and objective measures such as accelerometers are preferred when assessing physical activity levels. Furthermore, the cross sectional design of this study does not allow for the determination of causal relationships. Household physical activity may influence brain volume or brain volume may influence engagement in household physical activity. Future research examining the direction of this relationship is needed. For example, longitudinal studies assessing engagement in household physical activity and brain volumetric changes over time or interventions studies focused on increasing engagement in household physical activity. Aside from the mentioned limitations, strengths of this study include objective measures of cardiovascular risk, the use of advanced neuroimaging techniques, and the assessment of household and recreational physical activity separately.

In conclusion, household physical activity was positively associated with brain volume, specifically total gray matter volume in our sample. This is the first study to highlight associations with previously unexplored components of total physical activity and contributes to the growing body of literature guiding physical activity recommendations for older adults.

## Data Availability

The datasets used and/or analyzed during the current study are available from the corresponding author on reasonable request.
